# Cyclosporine-induced childhood generalized hypertrichosis^☆^^☆☆^

**DOI:** 10.1016/j.abd.2019.08.027

**Published:** 2020-03-19

**Authors:** Karilena Fernandes Souza, Paulo Fernando Barbosa de Camargo Andrade, Flávia de Freire Cassia, Maria Cristina Ribeiro de Castro

**Affiliations:** Department of Dermatology, Hospital Federal da Lagoa, Rio de Janeiro, RJ, Brazil

Dear Editor,

Hypertrichosis is defined as disproportionate hair growth in body areas with no action of androgen hormones and shows no effects of race, age, and gender.^1^ According to the disease onset age, it can be classified as congenital or acquired, and to the extension, as localized or generalized. Among the causes of acquired generalized hypertrichosis is the use of certain drugs, and the most frequently involved agents are phenytoin, cyclosporin, and minoxidil. Unlike hirsutism, hypertrichosis in prepubertal children is not related to underlying endocrinopathies, with the main triggering factor being adverse effects of medications.^2^

This case report highlights this association, illustrating an exuberant clinical presentation related to the use of Cyclosporin A (CsA).

A 6-year-old white female patient with a history of nephrotic syndrome for five years was using CsA because she had shown no improvement to corticosteroid therapy alone. There was a recent worsening of proteinuria, so pediatric nephrology decided to increase the dosage of CsA to 5 mg/kg/day. Three months later, the child developed a diffuse increase of terminal hairs on the face, trunk, limbs (Figure 1, Figure 2) and trichomegaly (excessive growth of eyelashes) (Fig. 3), and was referred to the dermatology department.

**Figure 1:**
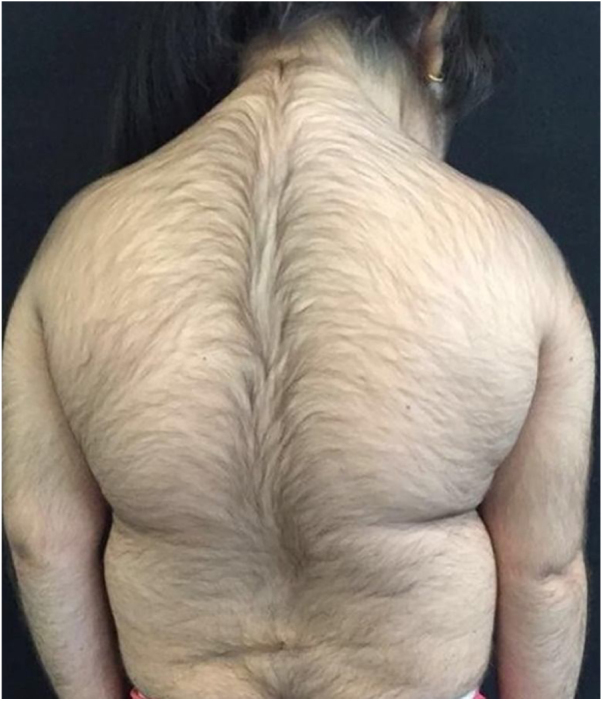
Hypertrichosis on the back and upper limbs.

**Figure 2:**
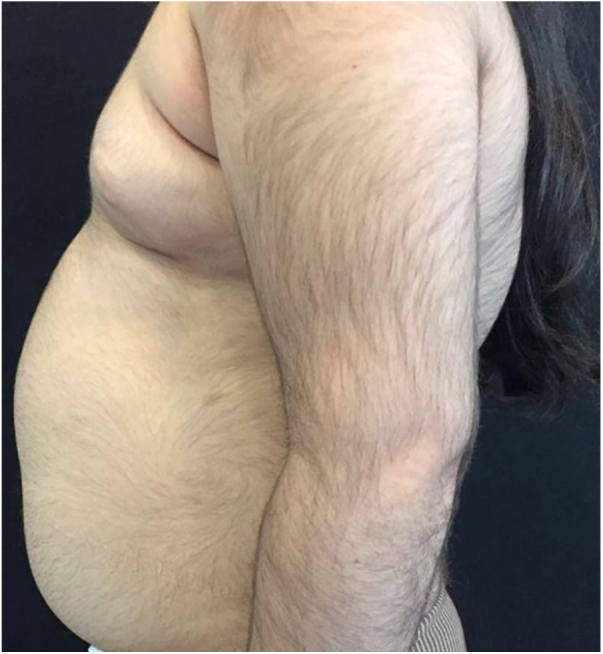
Diffuse growth of thick terminal hairs on upper limb and abdomen.

**Figure 3:**
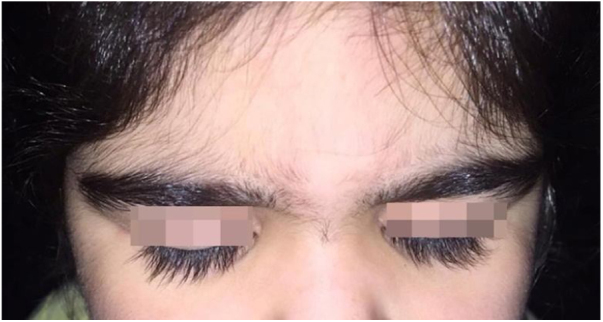
Ciliary hypertrichosis (trichomegaly).

The hormonal profile did not show abnormalities. Abdomen ultrasonography exam was also normal and the hirsutism hypothesis was discarded clinically and by laboratorial analysis. There were no ocular or oral cavity changes and the child's neuropsychomotor development was normal. However, the serum level of CsA was increased (467 ng/mL; Reference value: 150–300 ng/mL). These findings allowed the diagnosis for acquired generalized hypertrichosis related to Cyclosporin A. The proposed therapeutic measures included a reduction in daily medication dosage and temporary chemical epilation.

Cyclosporin is an immunosuppressive, calcineurin inhibitor whose mechanism of action is to selectively inhibit the function of the helper T lymphocytes (CD4+), consequently blocking the production of interleukins, especially IL-2.^3^ It is widely used in dermatology, including the treatment of psoriasis and atopic dermatitis^4^ and in other specialties, such as steroid-resistant nephrotic syndrome. Among the main adverse effects of dermatological interest are hypertrichosis, gingival hyperplasia, increased incidence of cutaneous neoplasia and infections.^5^

The pathogenesis of cyclosporin-induced hypertrichosis is not well established, but the literature describes that it is a dose-dependent side effect most commonly observed in children within the first six months of medication.^2^ Our patient had used CsA for a long time but developed generalized hypertrichosis specifically when a significant increase in cyclosporinemia was identified.

Xu et al. defend that CsA induces the anagen phase and inhibits the catagenic phase of the hair follicle and have demonstrated in animal models that the drug stimulates hair growth by promoting matrix cells proliferation, in addition to increasing the expression of growth factors such as Vascular Endothelial Growth Factor (VEGF).^4^ On the other hand, Ponticelli et al. attribute this adverse reaction to a possible induction in the alpha-reductase enzyme activity increase, responsible for the conversion of androgens to dihydrotestosterone in tissues.^5^

Other changes in the pilosebaceous unit such as sebaceous hyperplasia, keratosis pilaris, and acne are described and probably result from the elimination of the drug by the sebaceous glands because it is a lipophilic medication.^5^ However, the patient did not present cutaneous manifestations besides the exuberant hypertrichosis.

Drug-induced hypertrichosis is reversible with the suspension of the drug and may take from months to years to complete resolution, depending on the hair cycle of the affected area (face: about three months, arms about one year).^1^ Regarding this case, neuropediatrics chose to reduce the dosage of the drug until normalization of the serum level of cyclosporine was achieved, resulting in improvement of the skin changes, but we preferred to associate temporary chemical epilation until complete resolution of the dermatological changes since the child was suffering from bullying because of her appearance.

In renal transplant or nephropathic patients tacrolimus can be substituted for CsA, since both act similarly and tacrolimus causes fewer cutaneous abnormalities and does not trigger gingival hyperplasia.^5^

The reported case illustrates exuberant cyclosporin-induced hypertrichosis that, despite being a well-documented adverse effect, is poorly observed by dermatologists.

## Financial support

None declared.

## Authors' contributions

Karilena Fernandes Souza: Statistic analysis; approval of the final version of the manuscript; conception and planning of the study; elaboration and writing of the manuscript; obtaining, analysis, and interpretation of the data; effective participation in research orientation; intellectual participation in the propaedeutic and/or therapeutic conduct of the studied cases; critical review of the literature; critical review of the manuscript.

Paulo Fernando Barbosa de Camargo Andrade: Statistic analysis; approval of the final version of the manuscript; conception and planning of the study; elaboration and writing of the manuscript; obtaining, analysis, and interpretation of the data; effective participation in research orientation; intellectual participation in the propaedeutic and/or therapeutic conduct of the studied cases; critical review of the literature; critical review of the manuscript.

Flávia de Freire Cassia: Approval of the final version of the manuscript; elaboration and writing of the manuscript; effective participation in research orientation; intellectual participation in the propaedeutic and/or therapeutic conduct of the studied cases; critical review of the manuscript.

Maria Cristina Ribeiro de Castro: Approval of the final version of the manuscript; intellectual participation in the propaedeutic and/or therapeutic conduct of the studied cases; critical review of the manuscript.

## Conflicts of interest

None declared.
